# Transactions of the Medico-Chirurgical Society of Edinburgh, Vol. II. Continued from No. X. p. 408

**Published:** 1827-01

**Authors:** 


### *Transactions of the Medico-Chirurgical Society of Edinburgh.* Vol. II. with Plates.

(Continued from No. X. p. 408.)

---

### Art. I.

### *A Case of Purpura Hæmorrhagica terminating fatally, with the Appearances on Dissection.* By P. Fairbairn, M.D.

There are few diseases which occasion more perplexity and doubt in the mind of the practitioner than the one in question. He sees blood oozing from the mouth and other openings, while dark or livid spots are scattered over the surface, apparently indicating a broken down state of the vital fluid. Yet he is told by great authority, that the hæmorrhagic effort is of an active kind, and requiring depletion—while others, of no mean note, view the disease as one exemplifying a high degree of asthenia, and demanding the most effective tonics. It, therefore, requires some nerve, or some insensibility of nerve, to go coolly to work in such cases. We want farther evidence indeed, and a greater number of facts, to settle the pathology and treatment of purpura hæmorrhagica.

*Case.* On the 18th November, 1823, Dr. Fairbairn was called to a man, aged 24, of regular habits and robust constitution, but subject to vicissitudes of temperature, in his trade of book-binding. He complained of deep-seated pain in the left breast, aggravated by deep inspiration or coughing—breathing laborious, with sense of suffocation on attempt to stand—countenance flushed and anxious—copious discharge of dark venous blood from the mouth, apparently oozing from its mucous membrane, and partly expectorated from the lungs—numerous petechiæ and vibices on the arms, neck, and trunk, varying in magnitude from a mere point to the size of a sixpence. There were none on the hands or face. On the chest and leg, of one side, there were two large livid blotches resembling ecchymosis. These petechial spots were of various colours—bright red—purple—yellow, but not elevated. In the mouth, similar spots occupied the gums, cheeks, tongue, and fauces. The tongue was covered with a dark fur—urine of a grumous appearance—pulse 110, firm and sharp—some heat of surface—bowels loose. Reports that he experienced depression of spirits, lassitude in the limbs, pains in the head and chest, tickling cough, chilliness and flushes for several weeks previously. On the 16th November, the petechiæ made their appearance—on the 18th, he was seized with difficulty of breathing and fixed pain in the side. Dr. F. bled him to 26 ounces, which produced a disposition to syncope, and was followed by considerable relief. Blood not buffy—coagulum soft and tremulous. Fifteen drops of diluted sulphuric acid to be taken frequently in cold water. He had a restless night, from turbulent dreams; but the pectoral symptoms were relieved next day. Blood still continued to ooze from the mouth—and febrile symptoms were present. Eighteen ounces of blood from the same arm, and exhibited the same appearances. A dose of salts, which produced a fetid, loose stool. The next day he was again bled to 20 ounces, having shewn symptoms of determination of blood to the head. Syncope took place, and he expired on the morning of the 21st, the sixth day after the appearance of the petechiæ.

*Dissection.* The petechiæ appeared nearly the same as before death. The cellular and muscular textures on the neck and chest were injected with blood, and emphysematous. The thorax contained a pound of fluid resembling blood, of a dark colour and viscid consistence. The lungs contained a bloody serous fluid, and were less crepitous than natural. The bronchial tubes and trachea were filled with a similar fluid, and, beneath the internal coat of the latter, there was a slight effusion of dark venous blood. Dark blood was also effused into the anterior mediastinum, and between the layers of the pericardium. The heart was pale and flaccid; and, under its internal membrane, there was a similar effusion as in the trachea, giving a deep livid colour to the surface of the heart. The inside of the aorta presented an increased tint of redness, without evident thickening or change of texture. The floating viscera of the abdomen were of a dark leaden colour—there were a few petechiæ on the intestines—and the pyloric orifice of the stomach was studded with them. The liver was paler than natural, and somewhat softened—the spleen, when torn, effused a quantity of dark-coloured matter of a semi-fluid consistence. There were two large ecchymoses under the scalp—the brain and its membranes being healthy.

*Remarks.* This case is remarkable, as occurring in a stout muscular young man, and also on this account, that the shooting pains in different parts of the body, the general vascular excitement, the congestion and extravasation of blood into the mediastinum and other parts, afford strong grounds, in Dr. F.'s opinion, for considering the foregoing case of purpura as bearing a striking resemblance to active hæmorrhage, warranting the depletive measures recommended by the late Dr. Parry. It is questionable, as our author candidly observes, whether the depleting system was not carried too far, in this instance. But it is difficult to decide this point in the present state of our knowledge.

### Art. II.

*Observations on the Physiological Principle of Sympathy, chiefly in reference to the peculiar Doctrines of Mr. Charles Bell.* By W. P. Alison, M.D. F.R.S. E. Secretary to the Society and Joint-Professor of the Theory of Medicine in the University of Edinburgh.—(Transactions of the Medico-Chirurgical Society of Edinburgh, Vol. II.)

The question of the sympathy of the nerves has been so involved in obscurity from the want of *data* for discussion, that it has been almost abandoned by modern physiologists. Indeed it is obvious that, until we have attained a knowledge of the functions of the nerves, on which sympathy depends, little progress can be made in the investigation. During the times of Whytt, Haller, and Munro, this subject engaged the attention of a numerous class of writers, and was the source of many an ingenious and many an incomprehensible theory. In reviewing these theories, we observe that, although there was much abstruse and subtle reasoning employed in their support, yet the anatomy of the nervous system, as it was then taught, could never be brought to lend them much assistance. By some, the theories were retained, although the facts of anatomy were altogether neglected; and if there were others who sought to strengthen their views by reference to anatomy, they were accused of perverting it to suit their purposes. "Solent qui ita sentiunt, varias nervorum connexiones, maximè tum paris vagi, tum intercostalis nervi, quod latissimè pateant, studiose inquirere et ambitiose exaggerare, ut tum multis et tam variis sympathiis explicandis possint accommodari: sed operam manifeste ludunt et suo abutuntur otio."—*Astruc.*

When we mention the names of Dr. Whytt, Dr. Munro, and Haller, as having been engaged on this subject, it is sufficient to show that the "Principle of Sympathy" was not neglected. Yet these celebrated men have left it exceedingly vague and uncertain, principally because the consistency of their doctrines with the anatomy of the nervous system could not be proved: for no "Physiological Principle of Sympathy" can be satisfactory, unless it admits of an explanation on the grounds of the anatomy of the nerves. When we reflect on the imperfect knowledge which these authors possessed concerning the functions of the nerves, we cannot wonder at their having relinquished all hopes of reconciling their views with the facts of anatomy. Since their time, however, great improvements have been made in the knowledge of the system of the nerves: this division of anatomy has been converted from a state of intricacy and uncertainty into an open and fair field for legitimate inquiry.

And this leads us to remark how natural it was that Dr. Alison, while studying the "Physiological Principle of Sympathy," should refer to the recent discoveries of the functions of the nerves by Mr. Charles Bell; but we are astonished that he seems to have derived so little advantage from them. Mr. Bell's system of the nerves perfectly coincides with the opinions concerning sympathy which Dr. Alison supports in the above paper, as we shall presently be able to show; yet instead of perceiving this, he has perversely thrown aside anatomy, and, together with it, the only confirmation of his views. "The researches of Dr. Whytt and Dr. Munro on this subject were, as I think, quite successful in establishing two points in regard to such phenomena; first, that they cannot be explained by the connexions of the nerves of the sympathising parts; and, secondly, that they do not indicate any necessary consent or sympathy between the individual parts of the body; but, in general, are simply the effects of certain mental sensations." P. 170.

It may be seen from this quotation, that Dr. Alison does not seek for an anatomical explanation of the physiological principle of sympathy: indeed, he is so strongly convinced that sympathetic actions cannot be explained by the connexions of the nerves, that he has made this the grounds of an attempt to invalidate Mr. Bell's classification of the nerves.

In the investigation of this subject, the author of this paper has adopted metaphysical reasoning, in opposition to that which is grounded on anatomy: and he prefaces his observations by stating this opinion. "It is absolutely incumbent on every one who studies the physiology of the nervous system in the human body, to consider carefully the laws of the mental phenomena as made known to us by our own consciousness, and as generalized by the writings of metaphysicians." There is no one who can deny the justness of this sentiment; yet we think that, in studying the physiological question which he has chosen, there ought to be combined, likewise, proofs from anatomy, in order that sober and correct views might be attained.

The object of the first part of Dr. Alison's paper is to correct the idea of sympathy, implying a *necessary consent of the individual parts of the body with each other.* "If we take a systematic view of the different effects of mental acts on the bodily organs—not only of those which result from strictly voluntary acts, but of those, also, which result from instinctive efforts, and from sensations and emotions of the mind, we shall give, under these different heads, (but especially under that of the effects of sensations on the body,) what I believe to be a true account of most of the phenomena generally considered as indicating such necessary consent." P. 173.

It is his opinion, that sympathetic actions do not depend on any uniform consent of individual parts with one another, necessarily resulting from connexions among the nerves, but simply on the excitation of particular sensations; and he proceeds to illustrate his views by arranging the different kinds of sympathetic actions under the following heads:—1. The muscular actions, from irritation of distant parts, which are called sympathetic, appear to co-exist in the body only with the indications of sensation. 2. The attention of the mind cannot be fixed at one and the same time on two distinct objects: this is a proof that a mental sensation must necessarily intervene between the irritation of one part of the body, and the excitation of such movements as we have considered, in another. 3. In several instances of sympathetic actions, the sensations which are regarded as their cause may be excited by irritation of different parts of the body; proving that what is needful to the excitation of actions of this kind is not the irritation of particular parts, but the excitation of particular sensations. 4. Different irritations of the same parts do not excite the same actions if they excite different sensations.

Each of these statements is illustrated by numerous instances of peculiar sympathetic actions: and, after they have been duly discussed, the author proceeds to criticize Mr. Bell's classification of the nerves. "Mr. Bell appears, indeed, to consider his respiratory nerves as acting always under mental feelings, and so far his opinions agree with those of Dr. Whytt. But I think he has not observed, that several of the statements by which Drs. Whytt and Munro established the dependence of sympathetic actions on sensation, and their independence of connexions of nerves in their *course*, are equally conclusive against that dependence of certain sympathetic actions on the connexions of nerves *at their roots;* for which he himself contends." p. 191. In these sentiments we find an explanation of the reason why he refuses to adopt Mr. Bell's views: he has perceived that the class of respiratory nerves is made to account for several sympathetic actions, and being a disciple of Drs. Whytt and Munro, and convinced by them that the principle of sympathetic actions cannot be explained by the connexions of nerves, he has concluded that Mr. Bell's views are incompatible with their doctrine, and, therefore, that his explanation of the functions of the nerves must be unfounded. We shall presently shew that their views on the general principle are so far from being inconsistent with each other, that, if Dr. Alison had attended more carefully to Mr. Bell's classification of the nerves, he might have found that the facts of anatomy agree perfectly with the doctrine of Dr. Whytt. We must proceed, however, to notice the misconceptions on the part of Dr. Alison, which have been the cause of his opposition. We find that he details at great length, his objections to the classification of every individual nerve called respiratory. But it is to be remarked, that he attends almost entirely to the connexions which these nerves have at their roots, as noticed by Mr. Bell, and has neglected the great and important peculiarities to be observed in their distribution through the body. He says, in the end, at p. 222, that he has adduced sufficient statements to entitle him to affirm "that no bond of connexion has been ascertained at the roots of the nerves which Mr. Bell has designated as respiratory, which does not extend to other nerves not included, or not possessing any claim to be included in that system." He leads us to conceive that Mr. Bell founds his opinions of the functions of the nerves on the circumstance of their arising in a particular manner from certain columns of the spinal marrow and brain. Mr. Bell, on the contrary, was led to make his discoveries by attending to the parts of the body to which the nerves went: he observed that the class of nerves called respiratory took their course to those parts of the body which are, in function, connected with the organ of respiration: and that they were distributed there, in addition to another class of nerves, the properties of which he had discovered. He directed his attention to the phenomena exhibited in the apparatus for breathing: he ascertained that the endowments of the parts which constitute this organ are complicated in the same proportion as their nerves are numerous: he found that the excessive intricacy in the nerves of the face, jaws, throat, and breast, was on account of the individual nerves being agents of distinct powers—for combining the muscles in subserviency to different functions; and it was by carefully attending to each nerve, and viewing the actions of the muscles to which it is distributed, that he discovered its use, and was enabled to class it separately from the others. His views of the properties of the different *columns* were obviously deduced from knowing the functions of all the nerves which arise from them.

Let us here take an example, to prove that anatomy points out to us a difference between the nerves bestowing muscular power. For this purpose, let us select the portio dura of the seventh pair, to determine what is its function. The fifth pair of nerves supplies the same parts as the portio dura: the question, therefore, arises, what are the distinct offices of each? We know that the former is a compound nerve, resembling the spinal nerves, arising by two roots: the nerves proceeding from one root bestow sensibility to the head, while the other bestows power to the muscles of the jaw. The anatomical character of the fifth pair is, that it is distributed to almost every part of the head. It can even be traced most distinctly sending its branches to the muscles of the face. We have said that the fifth pair is a muscular nerve; yet these branches, which it sends into the muscles of the face, are not the motor nerves of the face: they possess no direct power of exciting these muscles to contraction. It would appear, from this alone, that the muscles of the face are of a class which require their functions to be bestowed by a nerve different from a common one. This leads us to observe what are the distinctions between the muscles of the jaws and those of the face, to ascertain whether their functions be different. The muscles of the face are subject to changes depending on the quiet or excited condition of the apparatus of respiration. Involuntary actions in the muscles of the eyelids, of the nostrils, and cheeks, are the consequences of the respiratory organs being violently roused. These muscles are much affected in certain passions of the mind, when we observe the whole respiratory organ moved. There is another peculiarity regarding these muscles: in certain conditions they are placed beyond the influence of the will, as in continued breathing, yet they are also subject to the will, as we observe in speech; and we must not omit to notice that the involuntary actions necessary for breathing proceed, at the same time that these muscles are employed, under the direction of the will, for other purposes. All these circumstances prove that the muscles of the face are endowed with properties which require a nerve different from a common nerve. Accordingly, we find that the portio dura, the respiratory nerve of the face, comes from a source in the brain different from that of the fifth pair; that, at its origin, it is close to other nerves, which may be proved to possess similar endowments with it; and that it takes a course different from the fifth pair, for the apparent purpose of combining its branches with the respiratory nerves. Are these not fair grounds for arranging this nerve (the portio dura) among a distinct class from the symmetrical nerves, and giving it a name corresponding to its character? Another conclusion to which Dr. Alison arrives exhibits to us the drift of his previous reasoning. "The nerves which Mr. Bell includes in this system (viz. the respiratory) are easily excited *by irritations not acting on any of their own number.*"

We conceive that *irritations* are conveyed by nerves of sensation, and not by motor nerves. The principal cause of his objecting to the system of the respiratory nerves, is founded on his not having separated in his mind the nerves of sensation from the nerves which command muscular action. Thus, because tickling the nostril excites the action of the muscles connected with respiration, he wonders why the branch of the fifth pair, within the nostril, is not included among the respiratory nerves. (P. 200.) The nerves of respiration are explained to be those which are distributed on a peculiar class of muscles, to control the actions connected with breathing. The nerve of the nose possesses an influence over the respiratory actions only in an indirect manner, by its connexion with the sensorium. If we desire to know which nerves produce the phenomena, we must look to the muscles which are called into action: these indicate which nerves were excited, because we know the nerves that go to supply these muscles. We should never think of looking among the nerves of *sensation*, to discover those destined to effect any *motion*. Here, then, appears to be the principal error of Dr. Alison: he has looked for respiratory nerves among nerves of sensation, when he might have known that the nerves of sensation form a distinct class in Mr. Bell's system; and he might have found that the fifth pair is included in that class. The peculiarity of the respiratory nerves consists in their being motor nerves, which supply the muscles employed in the actions of breathing, bestowing upon these muscles those peculiarities which distinguish them from all others, and combining them to act with one simultaneous effort.

It is necessary for us to remember that the respiratory nerves, in their course, are connected with nerves belonging to the other classes; so that the parts to which they go may appear to possess, through them, more endowments than one. If we find an organ, which is supplied by respiratory nerves, possessed with a peculiar sensibility, as the epiglottis, we are to ascribe the sensibility to the sensitive part of the spinal nerve, with which the respiratory nerve is bound up in one sheath; but the power of drawing the small muscles around the epiglottis into action, belongs to the respiratory nerve.

It is rather an interesting circumstance, that about the same time that Dr. Alison's essay appeared, Mr. Bell's paper on the nervous circle was published,* which contains opinions that coincide with those of Dr. Alison concerning the principle of sympathy. The views of Mr. Bell have been deduced altogether from anatomy, while pursuing his investigations into the functions of the nerves of sensation and muscular motion. But we have seen that Dr. Alison has totally rejected anatomy: and, on metaphysical grounds, has endeavoured to prove that the "peculiar doctrines" of Mr. Bell are incompatible with the "physiological principle of sympathy." We introduce the following quotation from the paper on the nervous circle to exhibit the author's views. It is his object to prove that, between the muscles and the brain, there is a circle of nerves: that one nerve gives the sense of the condition of the muscle to the brain: another conveys the influence from the brain to the muscle.

"For example; a patient of mine having, by a tumour pressing on the nerves of the orbit, lost the sensibility of the eye and eye-lids, she retained the motion of the eye-lids by the portio dura coming round externally, and escaping the pressure which injured the other nerves. Here the course of sensibility backwards to the brain was cut off, while the course of volition was free: she could not tell whether the eye-lid was open or shut, but, being asked to shut the eye which was already closed, she acted with the orbicular muscle, and puckered the eye-lids. When I touched the eye there was no winking, because the sensitive fifth pair had lost its power, although she could command the motion by voluntary exertion.

"In another instance, when the eye was insensible, touching the eye gave rise to a blush of redness and to inflammation, because the *part* was excited, but the muscles were not called into action: the relations which connect the sensibilities of the eye with the motions of the eye and eye-lid, are established in the roots of the fifth, and the seventh in the brain: the loss of function of the fifth nerve, therefore, interrupted the circle. Here, too, the motor nerve of the eye-lid was perfect, and the eye-lid readily acted under the influence of the will; but when the eye-lid was touched or pricked, it communicated no sensation. Is this insensibility of a motor nerve owing to the course of its influence being from the brain, and not towards it? When the nostril had lost its sen-

* In the Philosophical Transactions, 1826.

sibility from an affection of the fifth pair, we could not excite sneezing: when the tongue and cheek had lost sensibility, the morsel was permitted to remain between the tongue and the cheek until it was offensive, although the motions both of the tongue and of the cheek were perfect. All these phenomena correspond with the experiments on animals.

"Now it appears that the muscle has a nerve in addition to the motor nerve, which being necessary to its perfect function, equally deserves the name of muscular. This nerve has no direct power over the muscle, but circuitously through the brain, and by exciting sensation it may become a cause of action.

"Between the brain and the muscles there is a circle of nerves: one nerve conveys the influence from the brain to the muscle; another gives the sense of the condition of the muscle to the brain. If the circle be broken by the division of the motor nerve, motion ceases: if it be broken by the division of the other nerve, there is no longer the sense of the condition of the muscle, and therefore no regulation of its activity."—9.

Thus we observe that both gentlemen agree about the general principle—that a sensation conveyed to the mind, or the sensorium, precedes the action of a muscle: but there is an essential difference in the mode by which they arrive at this conclusion: one rejects anatomy altogether, while the other grounds his reasoning wholly upon anatomy, and is confined solely to the proofs which he derives from that source. In the paper alluded to, Mr. Bell has followed out this principle only so far as to show the connexion of the nerves of sensation with the muscles of voluntary action. Yet there is no reason assigned for confining the connexion to the voluntary muscles: on the contrary, the illustrations which we have quoted prove that the same principle applies equally to those which are involuntary. The nerve to which our attention is most frequently directed, is the portio dura, the motor nerve of the muscles of the face, and we know that this, at certain times, is under the control of the will, but most frequently is independent of it.

It is very remarkable, that, although Dr. Alison has stated his conviction, that sympathetic actions depend upon sensation, yet he has omitted to consider the nature and peculiarities of different sensations. Having arrived at the conclusion that there is a previous sensation of the mind, he conceives that he has attained the whole physiological truth concerning the principle of sympathy; and he has, perhaps, advanced so far as metaphysical research allowed. But it is to be considered,—1st. That sensibilities are infinitely varied over the whole body: 2ndly. That each kind of sensibility is attended with its appropriate sympathetic effects: and, 3dly,

That the nerves which convey sensation to the sensorium form a distinct class.

1. The nerves of sensation are distributed, as we well know, over the whole body: their most striking peculiarity is, that the sensibilities of different parts which are conveyed to the sensorium are infinitely varied. It was long a subject of controversy among physiologists whether certain structures in the body possess sensibility or not. But during the course of their inquiries they forgot to settle this question—whether the parts of the body are indiscriminately endowed with one kind of sensibility, or whether they are possessed, each with a peculiar kind, which is best suited for its protection? Both parties in this discussion appear to have fixed upon the sensibility of the integuments, as their test of sensation; for, whether they experimented upon the heart, lungs, intestines, muscles or tendons, they tried upon all the effects of those substances which we know cause pain when applied to the skin: and they judged of the degree of sensibility in each by the signs of pain which the animals exhibited. There can be no just comparison, however, between the sensibilities of different parts: by a wise provision of Nature, each organ is endowed with that kind of sensibility which is best suited for its protection and its offices in the economy. Thus, the common integument is not endowed with a sensibility which resembles that of the surface of the eye; the heart does not possess the same sensibility as the skin, yet it is most acutely sensible to its proper stimulus, —viz. the distention by blood; ligaments, tendons, and cartilages, possess sensibility of a certain kind, yet not such as to cause pain during the natural motions of the body; but, if a joint be stretched beyond its proper degree, its sensibility is immediately excited, as any one must know who has ever sprained his ankle.

More examples might be adduced to prove that sensations are different in the various structures of the body. They are implanted for the purpose of guarding the individual parts; and it is in conformity with this principle that each structure is endowed with an appropriate defence against injury.

2. Sensations, likewise, vary throughout the organs of the body, to produce certain combinations of the muscles.

If we consider the system of the animal body, we shall see many instances of peculiar sensibilities rousing certain muscles into activity. Irritation of the larynx, producing cough, is not a more curious example of a peculiar sensation stimulating an infinite number of large and small muscles, to combine for one purpose, than irritation produced by the pressure of the urine upon a particular spot of the bladder, in causing some muscles to relax, and others to contract, for the expulsion of the urine. In the muscles of the perineum in the male, we find another example, equally illustrative: for, when a different kind of sensation forms the excitement, a different combination of the muscles is produced. The arrangement in the action of the muscles for expelling the fæces forms also another illustration. We might multiply our instances by referring to the intestines, or to the heart, which contract on their contents, each acknowledging the influence of its proper stimulus. No example is more wonderful than that of the sensibility of the uterus, which, having remained inactive during gestation, at length contracts upon the fœtus, when the proper period for its expulsion has arrived.

These examples shew that certain sensibilities are implanted in various parts of the body, which, being excited, cause the actions of appropriate muscles. Each of these sensibilities is bestowed for useful purposes in the economy, and it is necessary that we should have a perfect acquaintance with the functions of a part, to understand the character of its proper sensibility.

3. Let us now consider the class of nerves which bestow sensation.

The anatomical fact, in relation to the variety of the sensibilities in the body, is this:—The nerves of sensation, which are those arising from the posterior column of the spinal marrow and the fifth pair, are endowed with sensibilities very differently modified. This may be best illustrated by considering the fifth pair. It is now ascertained that this nerve bestows, first, the degree of sensation which is peculiar to the skin; secondly, that acute sensibility which is possessed by the surface of the eye; thirdly, the sensation peculiar to the internal membrane of the nose; fourthly, that of the sides and tip of the tongue; and, fifthly, that of the muscles. Instances have occurred to prove that any of these sensibilities may be separately lost by affections of the fifth pair.

But, if we inquire further, we shall find that distinct sympathetic actions are dependent on each of these peculiar sensibilities, all of which are roused into activity by this individual nerve. 1. Dashing cold water on the skin of the face or head produces a deep inspiration. 2. The surface of the eye being irritated causes convulsive action of the orbicularis oculi, and draws tears from the lachrymal gland. 3. Tickling the inside of the nose excites the whole apparatus of the respiratory muscles to violent action, to produce sneezing; and in this case we may observe that the actions of a numerous class of muscles are arranged so as to direct and concentrate the expired air to sweep off the offending cause from the nostril. 4. Sapid bodies being applied to the tongue, cause an increased flow of saliva to the mouth, and draw into action the organs of mastication.

Cases have occurred* which prove that, when the fifth pair is destroyed, the sensibilities being lost, the muscles cease to produce their sympathetic actions; so that the eye may be pressed with the finger without causing winking, and the inside of the nose may be tickled without sneezing. Yet it is observed that patients, in these circumstances, can shut the eyelids, or draw the other muscles of the face into action, by voluntary effort.

It will now, perhaps, be allowed, that we have made a considerable step towards connecting anatomy with the "principle of sympathy." We may not, indeed, be able to explain why a certain kind of sensibility produces a certain sympathetic action, for that must depend upon the sensorium; but we have traced the individual nerve which conveys the *sensation* to the brain. Take sneezing, for example; we know that it is the fifth pair which receives the impression, and transmits it to the brain; and we further see that there follows the sensation, so produced, a peculiar excitement, drawing certain muscles into action.

It may be seen that, belonging to respiration, there are certain sensations which excite peculiar sympathetic actions, for the protection of this important function. It has not been asserted that the organs of respiration are peculiar in this respect: indeed, several instances have been stated, which show that the "principle of sympathy" extends over the whole body; and the only *peculiarity* of the respiratory apparatus is, that there exists a class of nerves destined for its functions, distinct from those which supply other parts.

If we attend particularly to the sensibilities which influence the act of respiration, we find that nature has guarded this function with a sensibility which is at all times awake. Under ordinary circumstances, it controls the complicated actions of breathing, without the cognizance of the will; and it becomes the object of our perception only when danger to the function is impending. No sooner, however, is this the case, than there is a feeling of anxiety excited, by which the respiratory muscles are sympathetically roused to their utmost exertion. We have frequent opportunities of observing the heaving of the chest and shoulders, the gasping and contractions of the throat, the spasmodic twitchings of the face, the energy that pervades

* See "Exposition of the Nervous System."

the whole frame, in those who suffer from obstruction in the windpipe, or die from hæmorrhage. The sensation seems to have its origin in the heart and lungs: being conveyed to the sensorium, it produces those phenomena through the medium of the respiratory nerves.

Through this sensibility, the heart and lungs are so combined, that, in all the variations to which they are liable, they are still found to correspond; the accelerated action of the one being directly followed by the excitement of the other. If there be an increased activity in the heart, it is necessary, for the circulation of the blood, that the apparatus moving the lungs should be accelerated in a corresponding degree: and, as the lungs themselves are passive, the influence must be sought for in those muscles which move the chest, the throat, and the nostrils.

"It is this painful sensation that introduces us to 'this breathing world,' which guards the vital functions through life as it draws us into existence. Pain is the agent which most effectually rouses the dormant faculties of both mind and body. While the child slumbers in the womb, it does not live by breathing, it possesses an organ which performs the office of the lungs. In the birth there is a short interval, betwixt the loss of the one organ and the substitution of the other: nor would the breath ever be drawn, or the lungs perform their function but for this painful and irresistible *viscus* which calls the whole corresponding muscles into action. Spasms and contractions are seen to extend over the infant's chest: the features are working and the muscles of the face agitated, probably for the first time: at last air is admitted into the lungs, a feeble cry is heard: the air in successive inspirations fully dilates the chest, and the child cries lustily: Now the regular inspiration is established, and the animal machinery subsides into repose."*

——————— "We came crying hither;
"Thou know'st the first time that we smell the air
"We wawle and cry: I will preach to thee, mark
"When we are born, we cry that we are come
"To this great stage of fools."—*Lear.*

In various powerful affections of the mind, there is a sensation referred to the breast, the existence of which is manifested by external signs. *Emotions* have been classed by writers on metaphysicks separately from the intellectual states of the mind, on account of the peculiar "vividness of feeling" which belong to the former. It would appear that simple consciousness is too dull a medium for these affections of the mind to be impressed through it alone; and there is added an excitement

* Essays on the Anatomy of Expression by Mr. C. Bell. *Second edition.*

of the frame, which gives intensity to the feeling. There is a certain turbulence produced in the action of the heart and the dilatation of the chest, and in the contractions of the throat and face, whenever the mind is intensely affected with emotion: thus, for example, in terror, the heart beats with force against the ribs; "there is a spasm on his breast; he cannot breathe freely; the chest is elevated; the muscles of the neck and shoulders are in action; his breathing is short and rapid; there is a gasping and convulsive motion of his lips, a tremor on his hollow cheek, a gulping and catching of his throat." The explanation of these phenomena seems to be this:—The nerves of respiration have been influenced by the condition of the mind to produce these actions; this disturbed state of the heart and respiratory organs is quickly referred to the mind, through that sensibility which we formerly said watches over them: and thus a sensation is produced, very nearly resembling that which accompanies threatened suffocation.*

This view of the matter illustrates how corporeal sensations are reflected to the mind, to render us more vividly alive to its emotions; and it also shows that the mind may rouse certain parts of the body into activity, independently of a preceding sensation. In these instances, sensation is not the cause, but the consequence, of the particular actions already produced in the body.

According to Dr. Alison, in adopting Mr. Bell's views,

"We should be obliged to suppose that there are bound up, in what is commonly called the olfactory nerve, one nerve destined for the perception of nauseous smells, and connected with the nerves of the abdominal muscles and diaphragm,"—(and the stomach, &c. if he supposes the act of vomiting;)—"one for the smells that cause faintness, connected with the nerves of the heart; and one for savoury smells, connected with those of the salivary glands. And in the fifth pair of nerves we must suppose that there are bound up, one nerve for the perception of cold applied to the face, and connected with the phrenic and intercostal nerves, by which the act of inspiration is performed, and also with the cutaneous nerves of the whole surface, by which a general constriction of the capillaries is produced; one for the perception of irritations of the nostrils, connected with the phrenic and lower intercostals and lumbar nerves, by which the act of sneezing is performed; and one for the perception of pain in the eyeball, cheeks, or teeth, possessing little or no connexion with the respiratory nerves; and so in other instances."

If, indeed, we expected to see all obscure phenomena ex-

---

* For further illustrations of this subject, the reader is referred to the second edition of Mr. Bell's Essays on the Anatomy of Expression.

plained,—the effects, for example, of odours giving pleasure or pain, or exciting disgust and nausea: if all those singular nervous affections, which are variously exhibited in different individuals, had been attempted to be accounted for, by this new system of the nerves, we should have thought of Mr. Bell's observations as Dr. Alison does. But we have remarked that he most resolutely avoids entering on subjects of this kind, which promise no successful results. He has limited us to the observation of certain facts: he has taught which nerves convey sensation from the part to the sensorium: he has shown by what nerves the impression proceeds from the sensorium to the muscles, combining them into simultaneous operation. But he has not attempted to explain anatomically what connexions are formed betwixt the roots of the sensitive nerves and the muscular nerves, in what is called the sensorium. We have heard Mr. Bell propose it as a question in his Lectures, how far the motions of parts, excited by titillation or irritation, depend upon the connexions of the nerves in the body, and how far on the connexions of the nerves in the brain. For example, when a creature continues to breathe without its head, or when an acephalous fœtus breathes without cerebrum or cerebellum, is there not a connexion between the heart and lungs and the muscles of respiration, without the intervention of the brain? He illustrated this question by mentioning, on the other hand, the muscles of the eye acting under the irritation of the eye, as a proof that the brain forms a part of this circle of relations; but the excitement of the heart and the intestines, contracting upon being distended, as other instances of the connexion being formed without the intervention of the brain. Dr. Alison is not aware of the care with which Mr. Bell has impressed in his Lectures, that there is a whole system of nerves, of which the powers are not yet ascertained,—those called the *sympathetic*, which may at some future time be made to resolve these difficulties.

It is only by observing the actions of certain parts of the body, and then by referring to the ascertained functions of the nerves which supply these parts, that we can distinguish which nerves were influenced directly by the sensorium in producing these actions: it therefore appears that so long as our acquaintance with the functions of the nerves is imperfect, we shall experience difficulties in accounting for sympathetic actions. Thus, recurring to the instance of irritation of the eye, it was noticed that, besides the spasmodic closing of the eye-lids, there was an increased secretion from the lachrymal gland, and a blush of inflammation on the conjunctiva. It was not attempted to explain by which nerves the two latter circumstances were effected; because physiologists have not yet ascertained which nerves proceeding from the brain possess an influence over the secreting and the circulating organs. At present it is only stated by Mr. Bell as a conjecture that the sympathetic nerve controls these functions. But, in explanation of the winking of the eye, we were enabled to say, that the portio dura caused this, because we know it is the function of that nerve to command the muscles of the face.

We, who are pleased with the simplicity and the interest which these views give to the nervous system, may be allowed to express a regret that an ingenious physiologist, conversant with many of those authors who have written on the *theories* of the nerves, should present us their *anatomy* in the same intricacy with which it has been hitherto enveloped.

Let us mark the advantages which the physiology of the nerves has derived from the strict attention which Mr. Bell has paid to the facts of anatomy in his late investigations. First, he has shown the composition of the spinal nerves; that the whole range, arising from the spinal cord, having ganglions upon their roots, are sensible nerves; and that the anterior roots are muscular nerves: Secondly, that the fifth pair in the head is of the same composition with the nerves of the spine: Thirdly, that, as this nerve corresponds in structure with the spinal nerves, so it corresponds in function: Fourthly, he has distinguished the nerves of the face into two classes, and shown that they possess distinct endowments: Fifthly, he has proved that there is a set of nerves exclusively distributed upon the muscles of respiration: Sixthly, he has shown, by comparative anatomy, that these nerves do not exist, except when the act of respiration is to be performed:—These form altogether a most brilliant proof of the importance of anatomical investigation.

We have heard some gentlemen say that these new views are very ingenious and very curious; but they have asked, of what use are they in the practice of medicine and surgery? We may reply, that it is impossible, in the early stage of any discovery, to foresee how far its influence may extend. But is it nothing to have proved that palsies, which were supposed to depend on the condition of the brain, may arise from local causes, quite independent of that organ, and comparatively of little danger?—To have pointed out to the surgeon the difference between a motor nerve and one of sensation,—and the consequent impropriety of cutting them indiscriminately in cases of tic douloureux, as formerly? Will any surgeon, who is acquainted with these new views, in operating on the face, divide the portio dura, in the expectation that its functions would be performed by branches of the fifth pair? Or would he hazard the destruction of the eye, when operating on tumors of the face, by unnecessarily dividing those branches of the portio dura which pass to the eyelids?

In conclusion, we would say that it is to be regretted, with regard to papers such as we have been endeavouring to criticise, that the general reader, who may not, perhaps, be very intimately acquainted with the subject, is more apt to rely on the character of the writer, than to weigh critically the value of his arguments.

### Art. III.

*A Case of Empyema, successfully treated by Paracentesis Thoracis.* By James Pitcairn, M.D. Fellow of the Royal College of Surgeons.

*Case.* Susan Smith, aged 11½ years, had been a healthy child, but had received a fall on her left side, a week before the date of report, by which some of her ribs were supposed to be hurt. On the 19th, (month not stated) there came on acute pain in the side, with fever, cough, and quick breathing. On the 20th, Dr. P. saw her, and found the difficulty of breathing amounting almost to orthopnœa—great pain in the side, tickling cough, hard, full pulse, flushed face. She was bled to 14 ounces, by which she was much relieved. Purgatives and antimonials. The relief was but temporary—the bad symptoms returned—she was again bled to syncope, with immediate benefit. But the inflammatory symptoms often returned, and in spite of antiphlogistic measures, a deep-seated pain remained in the thorax, with cough and short breathing, aggravated by exposure to cold air. The pulse kept uniformly above 110, ranging to 130. In the course of six weeks she was much emaciated—had constant cough, with copious expectoration—frequent diarrhœa—and hectic paroxysms. She was sent into the country, but soon returned unbenefited. Six months after this Dr. P. was sent for, on account of the extreme urgency of the cough, profuse expectoration—difficulty of breathing—livor of countenance. On examination, the left side of the chest was found to be very much enlarged and prominent in all directions. "The *base* of the heart was found pulsating under the *right* nipple, and its *apex* about 2½ inches more to the right." The action of the organ was regular, but very rapid, 145 in the minute—respiration short, rapid, and difficult.

After some hesitation an operation was consented to; an incision was made between the 5th and 6th ribs, and a flat trochar introduced into the thorax, by which 18 ounces of thick pus were evacuated, with great relief to the breathing and cough. The size of the left side of the chest was thus much diminished, and the pulsation of the heart returned to near its natural situation. On the second and third days, three and a half pounds of pus were withdrawn. During the six following days there was very little discharge, and, as the breathing was getting more embarrassed, a larger trochar was introduced, and 3½ pounds of pus evacuated. Soon after this the patient was examined by Dr. Cullen, when the following stethoscopic report was made.

" 'The whole of the left side of the thorax was greatly enlarged; the intercostal spaces prominent; and the angles of the ribs of greater dimensions than on the opposite side. Percussion, wherever practised, occasioned an uniform dull sound; there was, in fact, a total want of sonorosity. The heart was found pulsating on the right side. The stethoscope could not detect respiration under the clavicle, nor in the præcordial nor lateral regions; and it was heard in the back only, for the space of about an inch and a half, close to, and all along, the vertebral column. Respiration was heard but very feebly in the highest part also of the lung, viz. in the supra-clavicular fossa. From these symptoms it was evident, that the lung, pressed by the large quantity of superincumbent fluid, was adhering close to the spine, and to the upper part of the thoracic cavity, and consequently its tissue must have been greatly condensed.' " 233.

We need not pursue the details of the case. The discharge continued for a long time, but ultimately decreased, and finally ceased. In October 1824, she was seen by our author in the enjoyment of good health. The left side of the chest, formerly much enlarged, is now much contracted, as described and figured by Laennec.

### Art. IV.

### *Case of Carditis, with unusual Symptoms; and the Appearances on Dissection, Death having occurred from another Cause.* By John Gairdner, M.D. &c. &c.

*Case.* Mr. G. aged 28, a medical gentleman, was suddenly seized on the 16th March, 1823, with a violent palpitation of the heart. An hour had elapsed before Dr. G. arrived, and the action of the organ had, in this time, greatly subsided, but was still preternaturally violent and rapid, being more than 120 in the minute. Complained of throbbing in his temples, and head-ach. He was so persuaded that any exertion would renew the attack, that he dared not stir. He had previously experienced a paroxysm, similar in kind, but much less violent in degree. Thirty ounces of blood were immediately abstracted from the arm, which relieved both the throbbing and head-ach. The pulse came down to 108—blood buffy. Saline medicines for the night. 17th. Lay quiet in bed, without palpitation, pulse 100, but quite unable to exert himself—even to change his position. In the evening, another fit of palpitation came on—followed the same course—abated considerably before Dr. G. could arrive—and was succeeded, as before, by distressing sense of throbbing on the temples. Bled again profusely —and again felt relieved—blood buffed. 18th. Had another attack in the evening of this day, though not quite so violent. Another copious bleeding—blood buffed as before—another exemption from palpitation. 19th. Dr. Thomson saw the patient. Leeches to the head. No attack till the evening of the 20th, but it continued twice as long as the former ones. After this he had one or two slighter attacks, which did not require any active measures. His chief distress now proceeded from inability for exertion. When he lay quiet he was easy, and had a soft natural pulse; but if he attempted to raise his body, his pulse instantly became very frequent, and painful sensations plainly evinced the danger of continuing the experiment.

"I had various opportunities afterwards, at different dates, of ascertaining the remarkable effect produced upon his circulation, by changes of posture. I have found him reclining in bed, with his pulse, as usual, quite natural; the next moment, after he had slided, in the gentlest manner, over the edge of his bed, so as to rest his feet on the ground, I have found it at 130; again, after he had resumed his original position, I have found it almost immediately down to 80 or 76.

"From this singular state he recovered slowly but progressively. He rode out frequently in a carriage, in which he reclined on a couch; and, by the end of the summer season, he was able to sit upright, and to walk without assistance. But a new train of symptoms now arrested my particular attention. Soon after the commencement of his illness I observed that he coughed occasionally. He had been liable, long before, to protracted colds, for some of which I had prescribed; and they had always yielded to medical treatment. On this occasion his cough was so slight at first as to arrest no particular attention, till it became increased, by an accidental exposure in the month of July. He expectorated a little, and became emaciated. In short, we suspected incipient phthisis; and the result justified our unfavourable anticipations. It is sufficient to say, that appearances became daily less equivocal, and that the case terminated fatally on the 7th November." 241.

On dissection, the lungs were found tuberculated throughout on both sides, the tubercles having passed into suppuration in different places. There was a large abscess in the left lung.

"Near to the apex of the heart we found a layer of dense organized lymph, closely investing a part of the parietes of both ventricles. On attempting to separate a portion of this layer, it was found to be firmly united to the substance of the organ, dipping between its muscular fibres, in the form of dense cellular tissue. It was obviously the result of previous inflammatory action, which had subsisted nearly eight months before, when he had the violent fits of palpitation. There was no adhesion of the heart to the pericardium. The valves, and every other part of the heart, as well as the great arteries, were perfectly healthy." 242.

The above is an interesting case, as shewing one of the many anomalous forms, which inflammation in, or contiguous to, the heart, assumes. There can be no doubt that inflammation, long previous to death, was the cause of the appearance on the surface of the heart, and as little doubt can be entertained that the time of its occurrence was that of the distressing phenomena detailed. But such is the versatility of character appertaining to this disease, that it would be very dangerous to consider a similar train of symptoms, in another patient, as indicating with certainty the same pathological condition of the organ. We have seen instances where inflammation of the surface of the heart went to a far greater extent than in the above case, and where the function was so little disturbed, the while, as to occasion little alarm in the mind of either patient or practitioner, till near the close of the scene. On the other hand, we occasionally witness such commotion of function in the organ of the circulation, from mere nervous sympathy, as alarms the practitioner excessively, and leads to an ultra-depletion that is at least unnecessary, if not injurious. These circumstances borne in mind, will make the practitioner very cautious both in his conclusions and practice, in diseases of the heart. We think that the plan adopted by Dr. Gairdner, in the present instance was very judicious.

### Art. V.

*Case of Hydrocephalus Chronicus, with some unusual Symptoms and Appearances on Dissection.* By Charles Miller, Surgeon. Communicated by Dr. Robertson.

This boy when only eleven months old, was observed by his mother to require his head-dress to be enlarged every three or four weeks, the progressive increase of size continuing regularly until about two years before his death, when the head measured 31 inches in circumference. There was no farther increase afterwards. His general health, for many years, was good, and his growth natural, except the head. He was latterly emaciated—his features corresponded with the size of the cranium. For the last two years there had been an oozing of water from his nose, to the extent of one or two dram glassfuls daily. This water was clear, and of greater specific gravity than common water. On keeping, it became muddy. His memory was particularly correct, and his mental faculties unimpaired.

*Dissection.* On the removal of the scull-cap an immense rush of water took place, until a wash-hand basin was completely filled, and a pint and a half run over. The basin contained seven pints. A small quantity of pus was found in the posterior lobe of the cerebrum of the left side, and the anterior portion of the medulla spinalis was covered with a layer of purulent matter. The dura mater appeared as if torn in shreds, and that not during dissection, but from previous disease. It was quite separated from the interior of the scull, as far down as the circular incision is usually made, the bone being so far quite denuded, except the attachment along the longitudinal sinus, which was morbidly thickened. The convolutions of the brain were floating like loose intestine, and defied dissection. They were quite a gelatinous mass. The ventricles were like large pouches, and it was remarkable how very firm and cord-like the nerves felt amongst the general mass of devastation. The opening through which the water had distilled into the nostrils was a foramen, an inch above and to the right of the crista galli, having a direct communication with the nasal cavity. There was much bloody serum in the spinal canal. The boy was 16½ years old when he died.

Mr. Miller thinks this case offers proof that mind is much more independent of matter than is supposed by some. If Mr. Miller can shew us a case where there is a manifestation of mind without any brain, we will acknowledge that mind is *quite independent* of matter; till then, we must continue to believe that its manifestation is *entirely* dependent on matter, however bad that matter may appear on dissection. Every one has seen respiration carried on, and pretty well too, with half a lung. Here, Mr. Miller might say, is a proof that breathing is much more independent of the lungs than is supposed by some. But such argumentation is unworthy of notice.

### Art. VI.

*Notes of some remarkable Cases.* By James Molleson, M.D.

*Case* 1. *Stricture of the Ileum, and artificial Anus at the Umbilicus, &c. &c.*—Lexy Cuthbert, aged nine years, was attacked in January 1823, with colic pains and vomiting, after which a degree of bulimia prevailed, sometimes with little or no appetite. When brought home from school, in March of the same year, she had shiverings and tremors, succeeded by convulsive fits, which recurred five or six times. Besides these, she had fainting fits, often twice a day. There was general extenuation—derangement of bowels—diarrhœa. Soon after the colic attack above-mentioned, swelling, hardness, and pain of the abdomen came on, especially about the navel—and in April, prominence, tension, and great tenderness about the umbilicus. These symptoms were dispersed; but in August they returned, and an abscess burst, discharging pus and various portions of food. For upwards of 14 weeks this opening (about the calibre of a quill) discharged frequently a cup-ful of alimentary matter. Hectic fever and cough came on in this period, and the unfortunate patient gradually sunk.

On dissection, it was found that the cavity of the abscess communicated with the ileum, the adhesions of the peritoneum being firm and extensive. There was a stricture of the ileum, situated some inches above the cæcum, and capable of admitting only an ordinary quill. In the lungs there were found tubercles and adhesions.

The other two cases are not of material interest.

### Art. VII.

*Case of the High Operation of Lithotomy.* By George Ballingall, M.D. &c. &c.

*Case.* D. Meek, aged six years, a delicate boy, was admitted into the Infirmary of Edinburgh, for difficulty in making water (from infancy) and unconscious discharge of it in bed. On sounding, a stone was struck by Dr. B. though without any satisfactory noise being then elicited. During this and another examination, the fæces were passed, and the rectum was considerably protruded. By a subsequent examination, with the finger in the rectum, and the hand above the pubes, the stone was distinctly felt, and ascertained to be of very large size. After due preparation, the operation was performed in the following manner.

" 'A silver catheter was introduced into the bladder, and after drawing off the small quantity of urine which it contained, an attempt was made to distend the bladder by means of tepid water injected through the catheter; this, however, was very imperfectly accomplished, in consequence of the boy's violent struggles, and the forcible resistance made by the action of the abdominal muscles.

" 'An incision about two and a half inches long was now made through the skin and linea alba, terminating at the symphysis pubis; the fibres of the pyramidal muscles were then separated in the mesial line, and partly detached from their origin in the pubes. The handle of the catheter being now depressed, its point was made to project about three-fourths of an inch above the symphysis, and a sharp-pointed bistoury was then pushed into the bladder, immediately below the point of the catheter, and carried down towards the pubes, by which sufficient space was made for the admission of the finger. On its first introduction, nothing could be felt except the extremity of the catheter; but on turning the point of the finger upwards, the stone was felt impacted in the upper part, or fundus of the bladder, altogether above the brim of the pelvis, and wholly above the wound. Attempts were now made to seize the stone with the forceps, which repeatedly slipped, bringing away with them considerable fragments of the stone. It being obvious, that nothing was to be gained by prolonging the incision downwards, it was now dilated upwards, on the point of the finger; and the attempts to bring away the stone by the forceps, scoop, and fingers in the rectum, were persevered in for some time without success. The stone, however, was brought nearer to the external orifice, and retained in that situation by the finger in ano. No farther prolongation of the wound upwards (however desirable) could be attempted, the peritoneum having been already laid bare, and the intestine threatening to protrude. Under these circumstances, an attempt was made to gain a little space, by cutting transversely; and a few fibres of the bladder were divided in this direction on the right side, when the stone was extracted, with a moderate degree of force. A quantity of tepid water was now thrown forcibly into the bladder, and the fragments washed out. The edges of the wound were besmeared with oil, with a view of preventing, as much as possible, the insinuation and lodgment of urine in the cellular membrane. Two stitches were passed through the upper end of the incision, supported by a strap of adhesive plaster; its lower extremity was left open, covered only by a slip of simple dressing, and a compress of soft lint. An elastic gum catheter was lodged in the bladder, and the patient conveyed to bed, having been in all upwards of half an hour on the table. The stone was of an irregular plum shape; weighing, exclusive of many large fragments, thirteen drams and one scruple: measuring in its longitudinal diameter upwards of two inches, and in its transverse diameter one inch and a half.' The fragments of the external layer, which were put into Dr. Turner's hands for analysis, contain some uric acid, but consist principally of mixed phosphate of lime and ammoniaco-magnesian phosphate." 261.

In the evening he was found to have much tenderness over the abdomen, with quick pulse, dry tongue, and hurried respiration. Leeches were applied near the wound, and some aperient ordered for next morning.

Next day the abdominal tenderness was diminished, and the urine flowed both from the catheter and the wound. But, by mid-day, matters changed greatly for the worse, and he died at 8 o'clock in the evening.

On dissection, there were found some marks of inflammation on the peritoneum, and a little bloody serum in the abdominal cavity. The opening in the bladder was nearly two inches long. The upper part of this viscus, embracing the stone, was found enveloped with strong muscular fibres, converging towards a point a little below the superior extremity of the wound. At this point the coats appeared thickened, and the internal surface of the bladder was highly vascular, and of a soft flocculent appearance. Below the place where the stone was lodged there was some disposition to organic contraction. Dr. Ballingall makes several remarks on the above case, for which we must refer to the work. His reasons for preferring the high operation in this instance, were—the magnitude of the stone—the peculiar situation which it occupied—and the possibility of being foiled in extracting it by the lateral operation. He feels himself at a loss to account for the calculus having originally formed a lodgment for itself in the fundus of the bladder.

Appended to this paper is a short communication from Mr. Russell, giving the particulars of a curious case, where a stone was lodged in the superior fundus of the bladder, which grasped the whole of the surface, excepting a small portion, about half an inch in diameter, opposite to the transverse axis. The bladder was most contracted at this point, beyond which it became more expanded, and formed the principal receptacle for the urine. The stone passed through the narrow neck of the bladder to the length of about three fourths of an inch. The bladder was thickened and contracted, and, near the centre of the upper fundus, there was an aperture nearly half an inch in diameter: but the stone was so firmly grasped by the bladder, at this part, as to prevent the urine from escaping into the cavity of the abdomen.

### Art. VIII.

*A Case of Disorganization of the Stomach of an Infant, with Pathological and Practical Remarks; intended as a Supplement to a former Paper on the same Subject.* By John Gairdner, M.D.

In the second volume of the present series, page 170, *et seq.* we gave an account of Dr. G.'s former paper on the above subject, while reviewing the first volume of these Transactions. Since that period, Dr. G. has seen only one decided case; but in it, the symptoms were so marked as to enable him, at the first glance, to recognize the existence of the disease. The subject was a boy, who was weaned at 11 months old, and, on the fifth day afterwards, was seized with diarrhœa, the stools being at first copious, green, slimy, and becoming watery in the course of the next two days. On the third day vomiting came on, with great thirst. These symptoms continued till the sixth day, when Dr. G. first saw the patient. There was then great emaciation—countenance expressive of anxiety—pulse 120—skin hot—thirst urgent—fæces nearly natural in appearance. The indications of cure which seemed most proper to Dr. G. in this case were to allay irritation, and produce a quiescent state of the alimentary canal. The ingesta were ordered to be of the mildest kind, and small in quantity—the gums were divided over the two middle incisores—no medicine to be given. The next day (7th of the disease) the vomiting had ceased—the diarrhœa, thirst, &c. much abated—the child seemed disposed to sleep. The regimen (half a wine-glassful of arrow-root every six hours) to be continued. The warm bath, if the heat should return.—*8th day*:—All the symptoms still more favourable. The same regimen. *9th day*:—"All the morbid symptoms gone." The arrow-root as before. Dr. G. began to have some hopes of a cure; but on the following morning (10th day) the child had three dark fetid stools, solid, with some griping. This was the beginning of a diarrhœa, which was never afterwards thoroughly arrested. By the 15th day, it amounted to six stools in the twenty-four hours, and, by the 20th day, to 10 stools per diem. The child died on the 30th day of the disease. During this period he had vomited once or twice only; but he had lost all appetite, had great thirst, and was exceedingly fretful and restless. Paleness and occasional coldness of the extremities, with feeble pulse, indicated, from day to day an increasing languor of the circulation. The emaciation was progressive.

*Dissection*. Intestines more thin and translucent than usual. Some trifling fæcal matters in the jejunum and ileum, of a deep yellow hue. There was bile in the gall-bladder—mesenteric glands sound, as were the thoracic viscera. On examining the splenic extremity of the stomach, a semi-transparency and blueishness were observed at one point, which Dr. G. knew to proceed from disorganization of its texture, proceeding from within outwards. On examination of the interior, Dr. G. found all the coats perforated, except the peritoneal, which was so much attenuated as to give way under the pressure of a few drachms of fluid introduced into the stomach.

*Remarks*. We confess that this case surprized us a little. For Dr. G. informs us, that he recognized the nature of the disease on his first visit to the little patient, and "warned its parents of the impending danger." Yet, in almost the same breath, he lays down his indications of treatment on which he "rested his hopes of cure." Notwithstanding this impending danger, the child lived 24 days afterwards, and at one time in this period Dr. G. reported—"all the morbid symptoms gone." When we reflect on the symptoms described by Dr. G. as obtaining when he was first called in, we are really at a loss to know how he could, *by them*, recognize so formidable a malady as approaching disorganization of the stomach. Every practitioner meets with such symptoms daily, where no fatal issue takes place. Besides, when we recollect how quickly these circumscribed disorganizations of the stomach are produced, and how often they are seen in bodies, where no signs during life indicated their previous existence, we can hardly bring ourselves to believe that the affection of the coats of the stomach was the original cause of the symptoms. We are much more disposed to think that it arose in the course—perhaps near the close of the disease. The actual erosion, indeed, is considered by Dr. G. himself as a *post-mortem* process. "I am still of opinion that, whatever the changes may be which occur during life, the destruction of the stomach occurs after death, by the action of the fluids within upon its texture." The following observations respecting the subject of weaning, we deem worthy of insertion.

"1. *The condition of the Patient.*—If it has been liable to diarrhœa, or is a feeble child, emaciated by previous illness, weaning ought, if possible, to be avoided. If diarrhœa is actually present, the child should not on any account be weaned.

"2. *The season of the year.*—The disease seems to be most apt to happen in the hot weather of summer and autumn. In this the experience of Cruveilhier, and I think of Jaeger also, agrees with mine. Mr. Burns states, that the four instances of disorganization of the stomach of infants, which he found in the dissecting-room, and of whose history, during life, he was ignorant, were all in the summer season. Dr. Cheyne, whose cases of atrophia ablactatorum closely resemble mine in many of their characters during life (though he does not appear to have detected the same appearances after death), expressly states, that 'the disease chiefly arises in the autumn*.' I do not find that any cases, similar to that described above, have ever occurred in winter.

"3. *The Sex of the Patient.*—From the above case, and those registered in the table already referred to, it would appear that boys are more prone to the disease in question than girls. In some of the cases the sex has not been noted, but of eighteen in which it is noted, fourteen were boys and four girls." 337.

